# Editorial: Alternatives for the control of parasites to promote sustainable livestock

**DOI:** 10.3389/fvets.2022.1097432

**Published:** 2023-01-10

**Authors:** Wesley Lyeverton Correia Ribeiro, Vinícius Longo Ribeiro Vilela

**Affiliations:** ^1^Department of Physiology and Pharmacology, Universidade Federal do Ceará (UFC), Fortaleza, Ceará, Brazil; ^2^Department of Veterinary Medicine, Instituto Federal da Paraíba (IFPB), Sousa, Paraíba, Brazil

**Keywords:** animal health, parasitic diseases, endoparasite, ectoparasite, parasite control

Parasitic diseases are considered important limiting factors for livestock production worldwide, as they significantly compromise animal welfare and increase production costs. The degree of parasite involvement with livestock is related to several factors that involve the host–parasite relationship, namely, the genetic characteristic of resistance and resilience of the animals; nutritional status of the animals; level of parasitism; and the pathogenicity of the parasite populations involved. Furthermore, environmental conditions may be associated with a higher or lower level of occurrence of parasitic diseases, for example, in rainy seasons there is a greater probability of infections by gastrointestinal nematodes in ruminant animals.

Clinical signs of parasitism in production animals depend on the species of parasite involved in the infection and may include: anemia, lethargy, diarrhea, reduced food consumption, reduced weight gain and/or production of milk, infertility, in addition to high mortality rates in case of massive infections.

In traditional production systems, the mechanisms of animal infection, as well as the traditional ways to control parasites are already known which have provided effective and lasting protection to the animal population. Thus reducing animal management efforts and lowering costs of production. Currently, parasite control is performed, in most cases, with the use of synthetic ectoparasiticides and endoparasiticides. Traditionally, the main target of ectoparasites has been the control of ticks/mites and also, in some geographic areas, of flies/lice. Endoparasiticides such as benzimidazoles, imidazothiazoles, macrocyclic lactones and amino-acetonitrile derivatives (AADs) should control the main gastrointestinal nematodes. Other actives include triclabendazole, closantel and clorsulon for nematodes, and praziquantel and niclosamide for cestodes (1). Diagnostic methods with good sensitivity, specificity and aimed at making decisions are essential for choosing the appropriate parasiticide for use. The fecal egg count has used as the standard for monitoring infection levels. However, several automated diagnostic methods have been proposed for different species of parasites, including the detection of gastrointestinal helminth eggs. For example, Shaali et al. described magnetic properties present in helminth eggs that could be useful in the construction of innovative probes for the diagnosis of nematode species in real time.

Failures are common in the use of synthetic antiparasitic products for parasite control, consequently dosages are increased and the intervals between treatments are reduced, resulting in the acceleration of resistance development. Nematodes resistance to various anthelmints has been increasingly described around the world, demonstrating a worrying scenario, especially in tropical regions. Thus, alternative approaches to the control of nematode parasites in farm animals are necessary. Also, concerns of consumers are to reduce antiparasitic, drug residues in animal meat and in the environment.

While resistance should be considered as one of the main drivers of new innovation in antiparasitics drug development products with greater convenience are also important. For example, broad spectrum drug action, longer period of effectiveness of the active ingredient that requires a longer interval between treatments, pharmaceutical formulas with chemical stability and less toxicity are some characteristics to consider for a good antiparasitic. In this sense, the use of nanotechnology might be a good strategy to optimize the efficacy of drugs and to maintain the sustained effect of drugs at specific sites of action in the host (2). This area seems to be a fertile field for new advances in the control of parasites. Ali et al. presented a systematic approach to studies that have used nanotechnology to optimize the activity of substances with a potential anthelmintic effect on *Haemonchus contortus*.

Along with the use of antiparasitic drugs, to some extent, animal management strategies have also been successfully adopted. Knowledge about the life cycle of the parasites and the infection/infestation mechanisms enabled the development of efficient strategies for control, such strategies include pasture rotation, implementation of integrated crop-livestock, crop-livestock-forest systems, etc. While these strategies can be considered on many farms, they may not be a reality on some small farms.

In addition to the strategies already mentioned, examples of control alternatives are: nutritional management of animals, mineral supplementation, supply of vitamins, genetic selection of animals resistant to parasites, administration of vaccines, biological control (to control nematodes, for example), use of nutraceuticals and phytotherapy. It is noteworthy that these strategies have been extensively investigated by many who propose an integrated approach using as many strategies as feasible for control.

The great challenge of the coming decades for livestock will be to produce high quality food in an ethical, environmentally friendly and economically viable manner (3). Thus, studies aimed at reducing the use of antiparasitics, and leading to more sustainable control of parasites, should be increasingly encouraged. All this in a scenario where climate change can have direct and indirect effects on the epidemiology of important pathogens, in particular those that cause parasitic and infectious infections, with considerable repercussions on the health of farm animals (3, 4).

## Author contributions

All authors listed have made a substantial, direct, and intellectual contribution to the work and approved it for publication.
